# Outcomes of Peripheral Arterial Disease in Patients With Diabetic Foot Ulcers in Low- and Middle-Income Countries: A Systematic Review and Meta-Analysis

**DOI:** 10.7759/cureus.109752

**Published:** 2026-05-27

**Authors:** Tonny Acire, Shafie D Farah, Jackson Kakooza, Enock Mukiibi, Catherine R Lewis, Prosper Akankwasa, Sabastian Turyakira, Bienfait V Mumbere, Sedrick Bukyana, John Turyagumanawe, Musilim Abdulai, Maslah Osman Ali, Albert Ojangole, Michael Mugenyi, Frank J Ruhazwe, Benson Oguttu, Reuben K Nyaruga, Martin R Nkundeki, Olivier Iryivuze, Ali Abdikani, Innocent Ayesiga, Neel Rajendra, Samuel Oledo, Mohamed Abukar Nor, Evelyn N Balondemu, Theoneste Hakizimana

**Affiliations:** 1 Department of Surgery, Kampala International University, Western Campus, Ishaka, UGA; 2 Department of Surgery, St. Joseph's Hospital Kitovu, Masaka, UGA; 3 Department of Obstetrics and Gynecology, Kampala International University, Western Campus, Ishaka, UGA; 4 Department of Public Health, Hoima Regional Referral Hospital, Hoima, UGA; 5 Department of Surgery, Wentz Medical Centre, Kampala, UGA; 6 Department of Surgery, Kampala International University, Kampala, UGA; 7 Department of Community Health, Mbarara University of Science and Technology, Mbarara, UGA; 8 Department of Microbiology, Kampala International University, Western Campus, Ishaka, UGA; 9 Department of Clinical Psychology, Makerere University, Kampala, UGA

**Keywords:** amputation, diabetic foot ulcer, low- and middle-income countries, meta-analysis, peripheral arterial disease, prevalence, systematic review

## Abstract

Peripheral arterial disease (PAD) compromises tissue perfusion in diabetic foot ulcers (DFUs) and is a principal driver of amputation and mortality. The prevalence, risk factor profile, and clinical consequences of PAD in DFU populations specific to low- and middle-income countries (LMICs) have not been systematically synthesized. The aim of this study was to synthesize the available evidence on the prevalence, determinants, and clinical outcomes of PAD among patients with DFUs in LMICs. We conducted a systematic review and meta-analysis of observational studies reporting PAD prevalence, associated risk factors, or clinical outcomes among DFU patients in LMICs. Electronic searches of PubMed, Web of Science, Scopus, and Lens.org were supplemented by manual reference screening. Random-effects meta-analysis with restricted maximum likelihood (REML) estimation was applied. Risk of bias was assessed using design-specific Joanna Briggs Institute critical appraisal tools. Publication bias was evaluated by Egger's regression, Kendall's tau, and Rosenthal’s fail-safe N. Seventeen observational studies with 4,831 participants across nine LMICs were included. The pooled PAD prevalence was 43.00% (95% confidence interval (CI) 29.42%-57.74%; k = 11; N = 3,481; I² = 84.52%). Leave-one-out sensitivity analysis confirmed robustness of this estimate (range 39.37%-46.80%). Smoking was associated with more than twice the odds of PAD (pooled odds ratio (OR) 2.36; 95% CI 1.12-4.94; k = 6; I² = 61.9%), and hypertension nearly doubled the odds (pooled OR 1.84, 95% CI 1.20-2.82; k = 6; I² = 0.0%). PAD conferred a greater than fourfold increase in the odds of lower extremity amputation (pooled OR 4.14; 95% CI 2.24-7.63; k = 6; I² = 52.0%). Narrative evidence demonstrated reduced limb salvage, prolonged hospitalization, and excess mortality in PAD-affected cohorts. PAD is highly prevalent among DFU patients. Smoking and hypertension are consistent modifiable risk factors. These findings highlight the need for cardiovascular risk management within diabetic foot care in LMICs.

## Introduction and background

Diabetes mellitus is a growing global health challenge in both high-income and resource-limited settings [1,2]. One of its most serious complications, diabetic foot ulcer (DFU), is a leading cause of prolonged hospitalization, disability, and non-traumatic lower extremity amputation (LEA) [3,4].

Although infection and neuropathy are central to DFU pathogenesis, peripheral arterial disease (PAD) is particularly important because it compromises tissue perfusion, delays healing, and markedly worsens limb and survival outcomes [5,6]. In people with diabetes, PAD is often characterized by diffuse and distal atherosclerotic disease involving infra-popliteal and tibial vessels, which limits oxygen and nutrient delivery to ulcerated tissue and increases vulnerability to severe infection and tissue loss [7,8]. Its recognition in DFU is further complicated by coexisting peripheral neuropathy, which can mask ischemic pain and blunt classic symptoms such as intermittent claudication, resulting in delayed detection until advanced ulceration, gangrene, or critical limb-threatening ischemia has developed [1,3]. As a result, PAD in DFU is not only a vascular comorbidity but also a major determinant of prognosis.

These challenges are especially pronounced in low- and middle-income countries (LMICs), defined using World Bank income classifications [9], where late presentation, inconsistent vascular assessment, and limited access to specialized imaging, revascularization, and multidisciplinary foot care may worsen outcomes [4,10,11]. In such settings, DFU imposes substantial pressure on already constrained health systems, while delayed diagnosis of PAD may contribute to avoidable amputation, prolonged hospital stay, and excess mortality [12,13]. While the clinical burden is amplified in LMICs, the available evidence remains scattered across small and methodologically diverse studies.

Despite growing interest in diabetic foot disease, the literature on PAD among patients with DFU in LMICs remains fragmented. Existing studies have variably focused on prevalence, isolated risk factors, or selected clinical outcomes, with limited integration of these domains into a single evidence base [8,10]. To date, no systematic review has quantitatively pooled PAD prevalence, its associated risk factors, and its prognostic consequences specifically within LMIC DFU populations, leaving clinicians and health-system planners without a consolidated, region-relevant estimate to guide care. Moreover, most reports are single-center studies with heterogeneous case definitions, diagnostic approaches, and outcome measures, making it difficult to derive an overall estimate of PAD burden or to clarify which factors are most consistently associated with PAD and its downstream consequences in resource-limited settings. A focused synthesis is therefore needed to bring together evidence on prevalence, determinants, and clinical outcomes in LMIC populations specifically.

This systematic review and meta-analysis aimed to synthesize the available evidence on the prevalence, determinants, and clinical outcomes of PAD among patients with DFUs in LMICs. By integrating quantitative and narrative evidence across LMIC settings, this review sought to provide a clearer understanding of the burden of PAD in DFU, identify clinically relevant associated factors, and clarify its implications for amputation risk, limb salvage, and health-system burden.

## Review

Methods

Study Design, Protocol Registration, and Reporting Standard

This study was conducted as a systematic review and meta-analysis of observational studies examining the prevalence, determinants, and clinical outcomes of PAD among patients with DFUs in LMICs. The review was designed and reported in accordance with the Preferred Reporting Items for Systematic Reviews and Meta-Analyses (PRISMA) 2020 statement [14]. The protocol was prospectively registered in the International Prospective Register of Systematic Reviews (PROSPERO) before study selection and data extraction commenced (Registration: https://www.crd.york.ac.uk/PROSPERO/view/CRD420261354878).

Eligibility Criteria

Studies were eligible if they enrolled patients with DFUs and reported data on at least one of the following: prevalence of PAD, association between candidate risk factors and PAD, or association between PAD and clinically relevant outcomes such as LEA, mortality, limb salvage, or hospital resource use. Observational study designs were eligible, including cross-sectional studies, prospective cohort studies, retrospective cohort studies, and related comparative observational designs. Studies were excluded if they did not involve a DFU population, did not report PAD status or a PAD-related outcome, lacked sufficient extractable numerical data, were duplicate reports of the same dataset, or were reviews, editorials, case reports, conference abstracts without usable data, or non-original research articles.

Information Sources and Search Strategy

A systematic electronic search was conducted in Web of Science, PubMed, Lens.org, and Scopus for studies published from January 2010 to March 2026. The search was supplemented by manual screening of reference lists from eligible articles and relevant related papers. Search terms combined controlled vocabulary and free-text terms for diabetic foot ulcer, peripheral arterial disease, amputation, risk factors, prognosis, and LMIC settings using Boolean (AND, OR) operators. Gray literature and preprint repositories were not formally searched, and eligibility was restricted to peer-reviewed primary research articles. The complete database-specific search strategies are provided in Supplemental material 1.

Study Selection

All retrieved records were imported into a screening file and reviewed in two stages. First, titles and abstracts were screened against the eligibility criteria. Second, potentially relevant records underwent full-text assessment. Screening was performed independently by two reviewers. Disagreements were resolved through discussion and, where necessary, consultation with a third reviewer.

Data Extraction

Data were extracted independently by two reviewers using a standardized data extraction form. For each eligible study, the following information was recorded where available: first author, publication year, country, study design, sample size, PAD diagnostic method, PAD case count or prevalence, crude 2 × 2 contingency data, reported or derived effect estimates, and relevant clinical outcome data.

For studies contributing to quantitative synthesis, raw cell counts were extracted or derived to calculate odds ratios (ORs), log-transformed ORs, and corresponding standard errors (SEs) where necessary. For studies contributing to non-pooled analyses or narrative synthesis, data were extracted on peripheral neuropathy, diabetes duration, ulcer severity, amputation, limb salvage, mortality, wound healing, and hospital resource burden. Each unique study was counted once in the overall study inventory, even when it contributed data to more than one analytical component. Full characteristics of all unique included studies are presented in Supplemental material 2.

Outcomes and Analytical Framework

The primary outcome was the pooled prevalence of PAD among patients with DFUs in LMICs. The secondary meta-analytic outcomes were (i) the association between smoking history and PAD, (ii) the association between hypertension and PAD, and (iii) the association between PAD and LEA.

Additional reported factors, including peripheral neuropathy, diabetes duration greater than 10 years, and ulcer severity, were summarized narratively when formal pooling was not appropriate. Prognostic and health-system outcomes, including amputation, limb salvage, mortality, wound healing, and hospital resource burden, were synthesized narratively when reporting formats or outcome definitions were too heterogeneous for meta-analysis.

Risk-of-Bias Assessment

Risk of bias was assessed at the study level using design-specific Joanna Briggs Institute (JBI) critical appraisal tools. Studies included primarily to estimate PAD prevalence were appraised using the JBI Checklist for Studies Reporting Prevalence Data. Studies that prospectively or retrospectively followed cohorts to evaluate predictors of clinical outcomes, such as amputation or mortality, were appraised using the JBI Checklist for Cohort Studies. All other studies focusing on associations between candidate risk factors and PAD prevalence were appraised using the JBI Checklist for Analytical Cross-Sectional Studies.

Each checklist item was rated as “Yes,” “No,” “Unclear,” or “Not applicable,” and each study received an overall appraisal of low risk, moderate risk, or high risk of bias. Risk-of-bias assessment was performed independently by two reviewers, with disagreements resolved by discussion and consensus. The full study-level quality assessment table is provided in Supplemental material 3.

Statistical Analysis

Quantitative analyses were performed using Jamovi version 2.6.44 with the metafor package in R version 4.4. Random-effects meta-analysis with restricted maximum likelihood (REML) estimation was used for all pooled analyses. A random-effects model was selected a priori in preference to a fixed-effect model because substantial clinical and methodological diversity was anticipated across studies conducted in different LMIC settings, such that a single common true effect could not reasonably be assumed; REML was chosen for its approximately unbiased estimation of the between-study variance.

For the primary prevalence analysis, study proportions were transformed to the logit scale before pooling to stabilize variances and account for the bounded nature of prevalence data. Pooled estimates and confidence intervals (CIs) were then back-transformed to the natural prevalence scale for presentation. For association analyses, ORs were pooled on the log OR scale and exponentiated for interpretation.

Between-study heterogeneity was assessed using Cochran’s Q, I², H², tau², and tau. A pre-specified subgroup analysis by geographic region was performed for the primary prevalence outcome. Sensitivity of the primary prevalence estimate was assessed using leave-one-out analysis. Publication bias and small-study effects were evaluated using Egger’s regression test, Kendall’s tau rank correlation, visual inspection of funnel plots, and Rosenthal’s fail-safe N (FSN). All statistical tests were two-sided, and statistical significance was defined as p < 0.05. Where fewer than four studies were available, where compatible 2 × 2 data were incomplete, or where exposure definitions were not sufficiently comparable, findings were not pooled and were instead summarized descriptively in a structured narrative form.

Results

Study Selection

**Figure 1:**
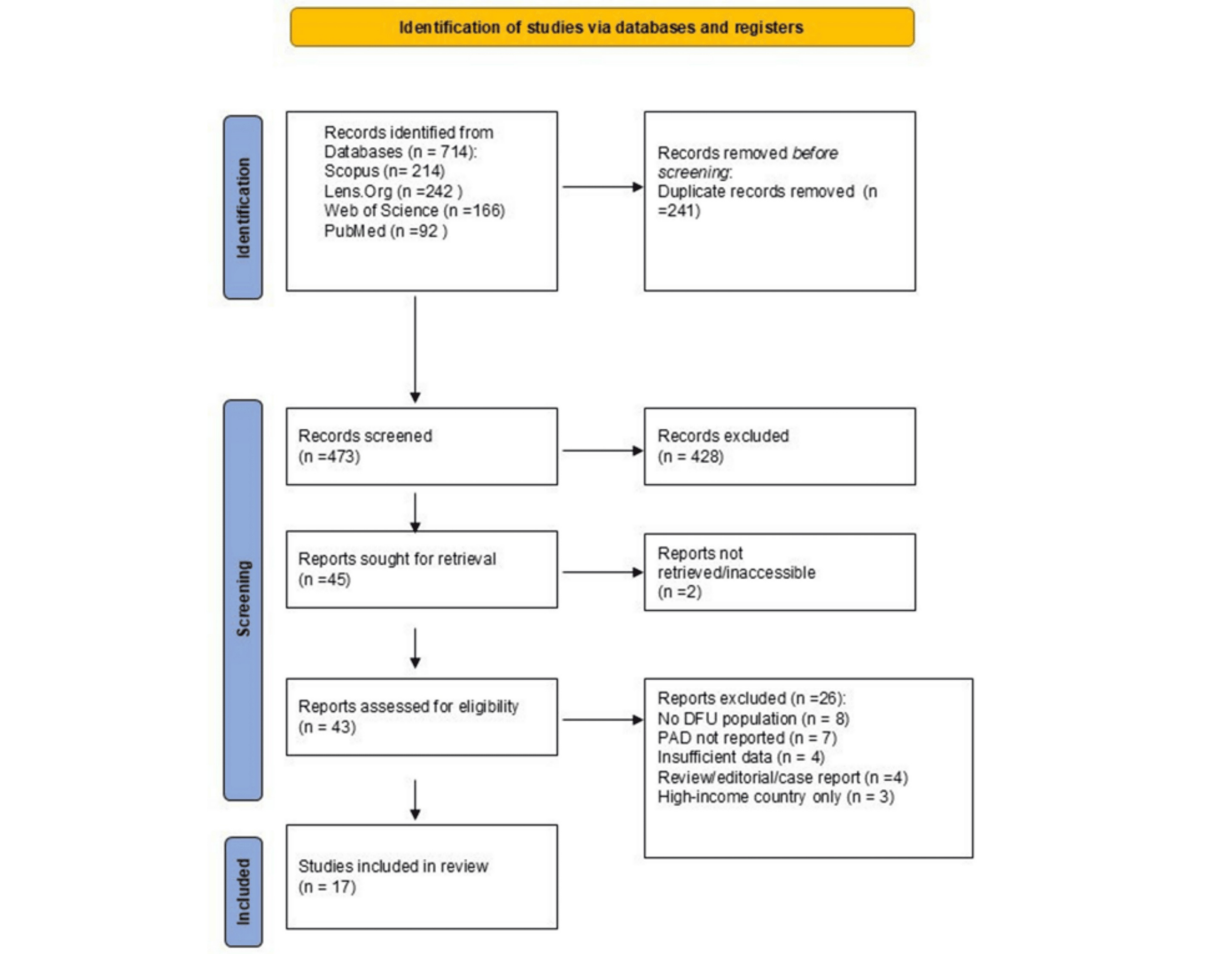
PRISMA Flow Diagram. Database searches identified 714 records (Web of Science = 166, Scopus = 214, Lens.org = 242, and PubMed = 92). After removing duplicates (n = 241), 473 records were screened, and 428 were excluded. Of 45 full-text articles, 2 were not retrieved. Among 43 assessed for eligibility, 26 were excluded, leaving 17 studies for review and meta-analysis. PRISMA: Preferred Reporting Items for Systematic Reviews and Meta-Analyses; DFU: diabetic foot ulcer; PAD: peripheral arterial disease

The systematic search of Web of Science (n = 166), PubMed (n = 92), Lens.org (n = 242), and Scopus (n = 214) identified 714 records in total. After the removal of 241 duplicate records, 473 records were screened based on title and abstract. Of these, 428 were excluded at the title-abstract stage, and 45 full-text reports were sought for retrieval. Two full-text reports could not be retrieved. The remaining 43 full-text reports were assessed for eligibility, and 26 were excluded (no DFU population, n = 8; PAD not reported or not measurable, n = 7; insufficient extractable numerical data, n = 4; review, editorial, or case report, n = 4; high-income country setting only, n = 3). Seventeen unique studies met the eligibility criteria and contributed data to at least one component of this review (Figure 1).

Of the 17 included studies, 11 contributed to the primary prevalence meta-analysis, six to each of the three secondary meta-analyses (smoking - PAD, PAD - LEA, and hypertension - PAD), and nine to the narrative synthesis of prognostic and health-system outcomes. An additional seven studies contributed data on peripheral neuropathy, diabetes duration, or ulcer severity that were reported narratively but were not formally pooled. Several studies contributed to more than one analytical component; the full contribution of each study is detailed in Supplemental material 2. No study was counted more than once in the derivation of the total.

Study Characteristics

The 17 unique included studies were published between 2010 and 2024 and collectively enrolled at least 4,831 participants, based on the minimum non-overlapping sample size reported across the 17 unique studies. Studies were conducted across nine LMICs in four geographic regions: Africa (Nigeria (three studies), Egypt (one study)), Asia (China (three studies), India (four studies), Bangladesh (one study), Jordan (one study), Iran (one study), and Pakistan (two studies)), and South America (Brazil (one study); Supplemental material 2). Study designs included prospective observational studies, retrospective cohort studies, and cross-sectional studies; design was not reported in the source material for five studies that contributed only 2 × 2 contingency data to secondary or non-pooled analyses. Most studies contributing to the primary prevalence meta-analysis used ankle-brachial index (ABI) < 0.9 as the primary PAD criterion; one Jordanian study used toe-brachial index (TBI) < 0.7 to define asymptomatic PAD, and several studies supplemented bedside vascular indices with Doppler, duplex ultrasonography, or computed tomography angiography (CTA) where clinically indicated (Table 1). PAD diagnostic methods were not reported in the source material for studies contributing only to secondary or non-pooled analyses.

**Table 1:** Characteristics of Included Studies in the Primary Prevalence Meta-Analysis. ABI: ankle-brachial index; CI: confidence interval; N: sample size; PAD: peripheral arterial disease; LMICs: low- and middle-income countries. 95% CIs were computed via logit back-transformation (delta method). Countries classified as LMICs per the World Bank [9].

Author (year)	Country	Region	Study design	PAD diagnostic method	N	PAD cases	Prevalence (95% CI)
Ikem et al. [3] (2010)	Nigeria	Africa	Prospective comparative	ABI < 0.9	46	31	67.39% (52.73%-79.29%)
Hao et al. [5] (2014)	China	Asia	Retrospective cohort	ABI < 0.9 + pulse palpation	1,002	702	70.06% (67.15%-72.82%)
Costa et al. [15] (2017)	Brazil	South America	Retrospective cohort	ABI < 0.9	654	160	24.46% (21.32%-27.91%)
Muthiah et al. [8] (2017)	India	Asia	Cross-sectional	ABI < 0.9 + Doppler	150	57	38.00% (30.59%-46.01%)
Ede et al. [13] (2018)	Nigeria	Africa	Cross-sectional	ABI < 0.9	60	37	61.67% (48.87%-73.02%)
Megallaa et al. [16] (2019)	Egypt	Africa	Cross-sectional	ABI < 0.9	180	36	20.00% (14.79%-26.48%)
Ravidas et al. [4] (2020)	India	Asia	Prospective observational	ABI < 0.9	105	80	76.19% (67.13%-83.37%)
Nobi et al. [11] (2019)	Bangladesh	Asia	Prospective observational	ABI < 0.9 + duplex	72	25	34.72% (24.67%-46.35%)
Abu-Jableh et al. [1] (2024)	Jordan	Asia	Cross-sectional	ABI < 0.9	133	38	28.57% (21.54%-36.82%)
Chen et al. [17] (2024)	China	Asia	Prospective cohort	ABI < 0.9 + Doppler	979	523	53.42% (50.29%-56.53%)
Kumar and Sethupathy [7] (2024)	India	Asia	Cross-sectional	ABI < 0.9	100	12	12.00% (6.94%-19.95%)
11 studies	9 LMICs	3 regions	-	All ABI < 0.9	3,481	1,701	12.00%-76.19% (range)

Risk of Bias

Risk of bias was assessed using design-specific JBI tools, including the JBI prevalence checklist for prevalence studies, the JBI cohort checklist for cohort studies, and the JBI analytical cross-sectional checklist for association-focused studies. Overall, the included studies demonstrated generally acceptable methodological quality, with adequate reporting of sampling procedures, PAD diagnostic criteria, and outcome ascertainment. Study-level ratings are presented in Supplemental material 3.

Primary Outcome: Prevalence of PAD Among DFU Patients in LMICs

**Figure 2:**
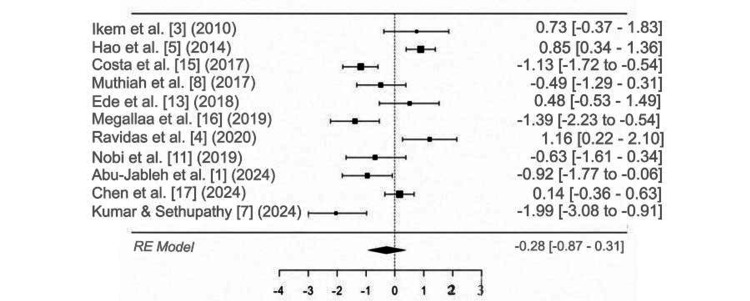
Forest Plot of Pooled Prevalence of PAD Among DFU Patients in LMICs. Forest plot displaying individual study prevalence estimates (back-transformed from the logit scale) with 95% confidence intervals (CIs) and the REML random-effects pooled estimate (diamond). Studies are ordered by geographic region. Square areas are proportional to inverse-variance study weights. Pooled PAD prevalence: 43.00% (95% CI 29.42%-57.74%). PAD: peripheral arterial disease; DFU: diabetic foot ulcer; LMICs: low- and middle-income countries; REML: restricted maximum likelihood

Pooled prevalence estimate: The random-effects meta-analysis (REML estimator; k = 11; N = 3,481) yielded a pooled PAD prevalence of 43.00% (95% CI 29.42%-57.74%), corresponding to a pooled logit estimate of θ̂ = -0.282 (SE = 0.303). Across included studies, approximately 43 in every 100 patients presenting with a DFU had concomitant PAD. The wide CI reflects the high between-study heterogeneity observed (I² = 84.52%). Table 2 presents the primary pooled estimate and geographic subgroup results. Figure 2 shows the corresponding forest plot.

**Table 2:** Primary Pooled PAD Prevalence and Geographic Subgroup Analysis (REML Random-Effects Model). CI: confidence interval; I²: between-study heterogeneity statistic; k: number of included studies; N: total sample size; PAD: peripheral arterial disease; REML: restricted maximum likelihood. ^†^South America subgroup comprises a single study [14]; the 95% CI was derived by applying the full-model between-study variance (tau² = 0.8204) to the single-study logit estimate, as between-study variance cannot be estimated at k = 1.

Analysis	K	N	PAD cases	Pooled prevalence	95% CI	I²
Primary pooled result - REML random-effects model (k = 11)						
Overall	11	3,481	1,701	43.00%	29.42%-57.74%	84.52%
Pre-specified geographic subgroup analysis						
Africa (k = 3)	3	286	104	47.80%	23.97%-72.67%	High
Asia (k = 7)	7	2,541	1,437	43.86%	28.22%-60.83%	High
South America (k = 1)^†^	1	654	160	24.46%	5.16%-65.86%	N/A

Geographic subgroup analysis: A pre-specified subgroup analysis by geographic region was performed. The Africa subgroup (k = 3; N = 286; PAD cases = 104) yielded a pooled prevalence of 47.80% (95% CI 23.97%-72.67%). The Asia subgroup (k = 7; N = 2,541; PAD cases = 1,437) yielded a pooled prevalence of 43.86% (95% CI 28.22%-60.83%). The South America subgroup comprised a single study from Brazil (k = 1; N = 654; PAD cases = 160), with a study-level prevalence of 24.46%. Because between-study variance cannot be estimated from a single study, the 95% CI (5.16%-65.86%) was derived by applying the full-model tau² (0.8204) to the single-study logit estimate and should be interpreted accordingly.

Secondary Meta-Analytic Outcomes

Three secondary random-effects meta-analyses were conducted to examine selected risk factors and outcomes associated with PAD in DFU patients. All analyses used REML estimation. Pooled ORs, 95% CIs, and heterogeneity statistics are presented in Table 3.

**Table 3:** Pooled Results of Secondary Meta-Analyses - ORs (REML Random-Effects Model). CI: confidence interval; I²: between-study heterogeneity statistic; k: number of included studies; OR: odds ratio; PAD: peripheral arterial disease; Q: Cochran's Q statistic; REML: restricted maximum likelihood All ORs are back-transformed from pooled log OR estimates via exponentiation.

Outcome	k	Pooled OR	95% CI	I²	Q (df)	p (Q)
Smoking history → PAD	6	2.3561	1.1219-4.9431	61.91%	12.54 (5)	0.028
PAD → lower extremity amputation	6	4.1371	2.2412-7.6293	51.97%	10.58 (5)	0.060
Hypertension → PAD	6	1.8404	1.1996-2.8236	0.00%	3.97 (5)	0.554

**Figure 3:**
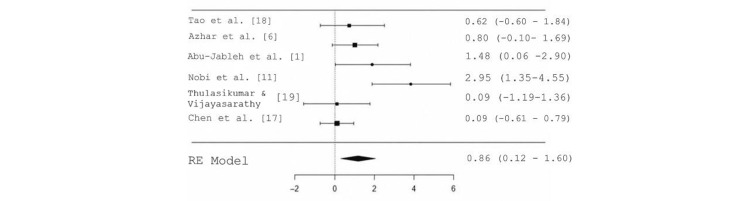
Forest Plot of Association Between Smoking History and PAD in DFU Patients. Forest plot displaying individual study log OR estimates with 95% confidence intervals (CIs) and the REML random-effects pooled OR (diamond). Pooled OR: 2.3561 (95% CI 1.1219-4.9431; p = 0.024). PAD: peripheral arterial disease; DFU: diabetic foot ulcer; OR: odds ratio; REML: restricted maximum likelihood

Smoking history as a risk factor for PAD: Six studies (k = 6; total N = 1,350) comparing PAD prevalence between DFU patients with and without a smoking history were pooled. Included studies were conducted in China [17,18], Egypt [6], Jordan [1], Bangladesh [11], and India [19]. The random-effects meta-analysis yielded a pooled OR of 2.3561 (95% CI 1.1219-4.9431; Z = 2.265; p = 0.024), indicating that DFU patients with a smoking history had more than twice the odds of PAD compared with non-smokers. Individual study ORs ranged from 1.09 [19] to 19.11 [11]. Moderate-to-substantial between-study heterogeneity was present (I² = 61.91%; Table 3). Figure 3 shows the forest plot for this analysis.

**Figure 4:**
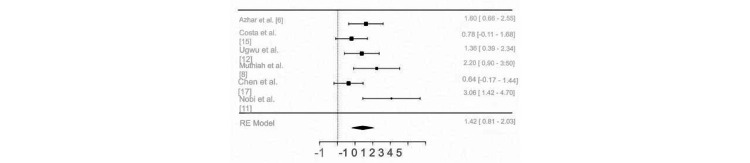
Forest Plot of Association Between PAD and Lower Extremity Amputation in DFU Patients. Forest plot displaying individual study log OR estimates with 95% confidence intervals (CIs) and the REML random-effects pooled OR (diamond). Pooled OR: 4.1371 (95% CI 2.2412-7.6293; p < 0.001). REML: restricted maximum likelihood; OR: odds ratio; PAD: peripheral arterial disease; DFU: diabetic foot ulcer

PAD as a predictor of LEA: Six studies reporting the association between PAD and LEA in DFU patients were pooled [6,8,11,12,15,17]. Individual study ORs ranged from 1.89 [17] to 21.39 [11]. The random-effects meta-analysis yielded a pooled OR of 4.14 (95% CI 2.24-7.63; Z = 4.54; p < 0.001), with the 95% CI lying entirely above 1.0. Moderate between-study heterogeneity was observed (I² = 51.97%; Table 3). Figure 4 shows the forest plot for this analysis.

**Figure 5:**
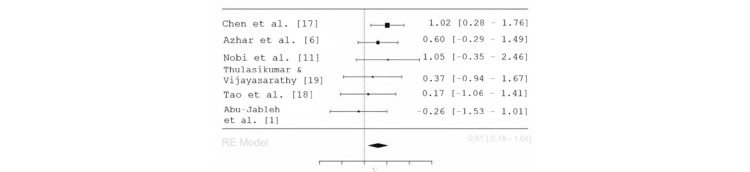
Forest Plot of Association Between Hypertension and PAD in DFU Patients. Forest plot displaying individual study log OR estimates with 95% confidence intervals (CIs) and the REML random-effects pooled OR (diamond). Pooled OR: 1.8404 (95% CI 1.1996-2.8236; p = 0.005). REML: restricted maximum likelihood; OR: odds ratio; PAD: peripheral arterial disease; DFU: diabetic foot ulcer

Hypertension as a risk factor for PAD: Six observational studies (k = 6; total N = 1,796) examining hypertension as a risk factor for PAD in DFU patients were pooled. Included studies were conducted in China [17,18], Egypt [6], Bangladesh [11], India [19], and Jordan [1]. The random-effects meta-analysis yielded a pooled OR of 1.8404 (95% CI 1.1996-2.8236; Z = 2.794; p = 0.005). Between-study heterogeneity was negligible (I² = 0.00%; Q(5) = 3.97; p = 0.554; Table 3). Because the six contributing studies were unevenly distributed across regions, including Asian, Middle Eastern, and African settings, regional subgroup findings should be interpreted cautiously and were not emphasized as definitive. Figure 5 shows the forest plot for this analysis.

Additional risk factors reported in eligible studies but not meta-analyzed: Several additional risk factors were reported by eligible studies but could not be formally pooled owing to an insufficient number of studies (k < 4), incomplete 2 × 2 data, incompatible exposure definitions, or heterogeneous measurement approaches. Findings for three domains - peripheral neuropathy, diabetes duration greater than 10 years, and ulcer severity - are reported narratively and summarized in Table 4.

**Table 4:** Additional Risk Factors Reported in Eligible Studies but Not Meta-Analyzed. A: PAD-positive and exposed; B: PAD-positive and unexposed; C: PAD-negative and exposed; D: PAD-negative and unexposed; ABI: ankle-brachial index; OR: odds ratio computed as AD/BC; PAD: peripheral arterial disease

Risk factor domain	Study (year)	Country	A	B	C	D	OR	Key finding	Reason not meta-analyzed
A. Peripheral neuropathy and PAD									
Peripheral neuropathy	Janbakhsh et al. [10] (2021)	Iran	95	25	44	32	2.76	PAD-positive patients had 2.76 times the odds of concomitant peripheral neuropathy	Insufficient k for pooling; heterogeneous neuropathy definitions
	Azhar et al. [6] (2021)	Pakistan	38	134	31	189	1.73	Moderate positive association	Incompatible exposure definitions across studies
	Thulasikumar and Vijayasarathy [19] (2017)	India	12	24	16	48	1.50	Positive association; smaller sample	Insufficient k for pooling
B. Diabetes duration > 10 years and PAD									
Diabetes duration > 10 yr	Hao et al. [5] (2014)	China	527	175	218	82	1.13	Weak positive association	Heterogeneous exposure thresholds across studies
	Janbakhsh et al. [10] (2021)	Iran	62	58	33	43	1.39	Positive association; overlaps neuropathy exposure	Insufficient k; incompatible exposure definitions
	Usman et al. [2] (2023)	Pakistan	62	58	41	45	1.17	Weak positive association, consistent direction	Insufficient k for pooling; incompatible exposure definitions
C. High ulcer severity/Wagner grade and PAD									
Ulcer severity/Wagner grade	Azhar et al. [6] (2021)	Pakistan	65	107	15	205	8.30	Strong positive association: highest severity grade strongly predicted PAD	Severity definitions differed across studies; formal pooling not justified
	Tao et al. [18] (2023)	China	21	57	4	38	3.50	Moderate-to-strong positive association	Incompatible grading scales
	Hao et al. [5] (2014)	China	373	329	144	156	1.23	Weak positive association	Incompatible grading scales; pooling not appropriate

Peripheral Neuropathy

Three eligible studies reported data on peripheral neuropathy as a binary exposure in relation to PAD [6,10,19]. Study-level ORs were 2.76, 1.73, and 1.50, respectively, all pointing in the direction of a positive association between neuropathy and PAD. Formal pooling was not performed because only three studies were available, and their neuropathy definitions were not sufficiently uniform to justify quantitative synthesis.

Diabetes Duration Greater Than 10 Years

Three eligible studies reported data on diabetes duration as a risk factor for PAD [2,5,10], with study-level ORs of 1.13, 1.39, and 1.17, respectively. All three ORs pointed in a positive direction, suggesting a modestly elevated risk of PAD among patients with longer-standing diabetes, although the magnitudes were small and the exposure thresholds were not identical across studies. Formal pooling was considered inappropriate owing to the small number of studies and incompatible exposure definitions.

Ulcer Severity and Wagner Grade

Three eligible studies reported associations between higher ulcer severity (Wagner grade) and PAD [5,6,18], with study-level ORs of 8.30, 3.50, and 1.23, respectively. The wide variation in ORs across studies is partly attributable to differences in grading systems used and in how severity thresholds were operationally defined. Formal pooling was therefore not performed.

Heterogeneity

Between-study heterogeneity was quantified using Cochran's Q test, the I² statistic, H², tau², and tau for all four meta-analyses (Table 5). Heterogeneity in the primary prevalence meta-analysis was high (I² = 84.52%; Q(10) = 61.61; p < 0.001; tau² = 0.8204). This is consistent with the broad variation in study-level prevalences (12.00%-76.19%) and likely reflects genuine differences in patient case-mix, study setting, and DFU severity across nine LMICs.

**Table 5:** Between-Study Heterogeneity Statistics - Primary and Secondary Meta-Analyses. H²: ratio of total to within-study variance; I²: proportion of total variance attributable to between-study heterogeneity (thresholds per Higgins and Thompson [20]: <25% negligible, 25%-50% moderate, 50%-75% substantial, and >75% high); Q: Cochran's Q statistic; REML: restricted maximum likelihood; SE: standard error of tau²; tau: between-study standard deviation; tau²: between-study variance; PAD: peripheral arterial disease

Meta-analysis	Q (df)	p (Q)	I² (%)	H²	Tau² (SE)	Tau
PAD prevalence - primary (k = 11)	61.61 (10)	<0.001	84.52	6.46	0.8204 (0.449)	0.906
Smoking → PAD (k = 6)	12.54 (5)	0.028	61.91	2.63	0.5061 (0.538)	0.711
PAD → amputation (k = 6)	10.58 (5)	0.060	51.97	2.08	0.2949 (0.366)	0.543
Hypertension → PAD (k = 6)	3.97 (5)	0.554	0.00	1.00	0.000 (0.175)	0.000

Heterogeneity was moderate-to-substantial in the smoking-PAD analysis (I² = 61.91%; Q(5) = 12.54; p = 0.028; tau² = 0.5061) and moderate in the PAD-amputation analysis (I² = 51.97%; Q(5) = 10.58; p = 0.060; tau² = 0.2949). In the hypertension-PAD analysis, between-study heterogeneity was negligible (I² = 0.00%; Q(5) = 3.97; p = 0.554; tau² = 0.000), indicating high consistency of effect across the six contributing studies.

Sensitivity Analysis

A leave-one-out sensitivity analysis was performed for the primary prevalence meta-analysis, iteratively excluding each of the 11 studies and re-estimating the pooled prevalence with tau² held constant at 0.8204 (Table 6). The pooled prevalence ranged from 39.37% (on exclusion of Ravidas et al. [4], which had the highest individual prevalence of 76.19%) to 46.80% (on exclusion of Kumar and Sethupathy et al. [7], which had the lowest individual prevalence of 12.00%). The maximum absolute deviation from the full-model estimate of 43.00% was +3.80 percentage points (on exclusion of Kumar and Sethupathy et al. [7]). No individual study exclusion changed the direction or clinical magnitude of the primary finding, confirming that the pooled estimate was not unduly influenced by any single study.

**Table 6:** Leave-One-Out Sensitivity Analysis - Primary PAD Prevalence Meta-Analysis. θ̂ = pooled logit estimate with the named study excluded; CI: confidence interval; pp: percentage points relative to the full-model estimate of 43.00%; SE: standard error; PAD: peripheral arterial disease Prevalence values were back-transformed using the inverse logit function. Tau² was held constant at 0.8204 throughout all iterations. The full-model row represents the primary analysis with all 11 studies included.

Study omitted	Pooled θ̂ (logit)	SE	Prevalence (%)	95% CI lower	95% CI upper	Deviation from 43.00%
Full model (k = 11)	-0.2820	0.3030	43.00%	29.42%	57.74%	-
Ikem et al. [3]	-0.3830	0.2931	40.54%	27.74%	54.77%	-2.46 pp
Hao et al. [5]	-0.4080	0.2947	39.94%	27.18%	54.23%	-3.06 pp
Costa et al. [15]	-0.2003	0.2946	45.01%	31.48%	59.32%	+2.01 pp
Muthiah et al. [8]	-0.2676	0.2942	43.35%	30.06%	57.67%	+0.35 pp
Ede et al. [13]	-0.3620	0.2935	41.05%	28.14%	55.31%	-1.95 pp
Megallaa et al. [16]	-0.1771	0.2941	45.58%	32.00%	59.85%	+2.58 pp
Ravidas et al. [4]	-0.4317	0.2938	39.37%	26.74%	53.60%	-3.63 pp
Nobi et al. [11]	-0.2546	0.2937	43.67%	30.36%	57.96%	+0.67 pp
Abu-Jableh et al. [1]	-0.2248	0.2941	44.40%	30.98%	58.70%	+1.40 pp
Chen et al. [17]	-0.3330	0.2947	41.75%	28.69%	56.08%	-1.25 pp
Kumar and Sethupathy [7]	-0.1281	0.2932	46.80%	33.12%	60.98%	+3.80 pp

Publication Bias

**Figure 6:**
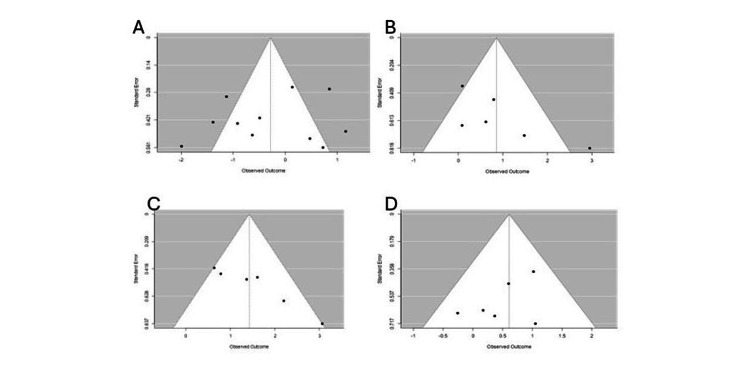
Funnel Plots of All Four Meta-Analyses. Panels show funnel plots for (A) primary PAD prevalence meta-analysis (k = 11), (B) smoking-PAD odds ratio (OR) (k = 6), (C) PAD-amputation OR (k = 6), and (D) hypertension-PAD OR (k = 6). The x-axis represents the effect size (logit-transformed prevalence or log OR); the y-axis represents the standard error. Asymmetry in panels (B) and (C) is consistent with a small-study effect. PAD: peripheral arterial disease

Publication bias was assessed for all four meta-analyses using Egger's regression test, Kendall's tau rank correlation, and Rosenthal’s FSN (Table 7). For the primary prevalence meta-analysis, neither Egger's test (intercept = -0.396; p = 0.692) nor Kendall's tau (τ = -0.018; p = 1.000) indicated funnel plot asymmetry, and visual inspection was consistent with symmetry (Figure 6A). The FSN of 6 reflects the proximity of the pooled logit estimate to zero - corresponding to a prevalence near 50% - rather than fragility of the pooled estimate; the formal asymmetry tests showed no evidence of publication bias.

**Table 7:** Publication Bias Assessment of All Meta-Analyses. FSN: Rosenthal’s fail-safe N; Kendall's τ: rank correlation between effect size and standard error (Begg's test); PAD: peripheral arterial disease. *FSN = 6 for the primary prevalence analysis; see text for interpretation. ^†^Statistically significant funnel asymmetry detected (p < 0.05). ^‡^FSN for k = 6 analyses with a conventional Rosenthal’s tolerance threshold of 5k + 10 = 40. FSN values below this threshold should be interpreted cautiously even when formal asymmetry tests are non-significant.

Meta-analysis	Egger's intercept	p (Egger)	Kendall's τ	p (Kendall)	FSN	Funnel asymmetry
PAD prevalence - primary (k = 11)	-0.396	0.692	-0.018	1.000	6*	Not detected
Smoking → PAD (k = 6)	+2.305	0.021^†^	+0.600	0.136	23	Detected^†^
PAD → amputation (k = 6)	+3.059	0.002^†^	+0.867	0.017^†^	93	Detected^†^
Hypertension → PAD (k = 6)	-1.182	0.237	-0.200	0.719	7^‡^	Not detected

For the smoking-PAD and PAD-amputation analyses, Egger's test identified statistically significant funnel asymmetry (p = 0.021 and p = 0.002, respectively), consistent with possible small-study effects. The smoking-PAD FSN was 23, below the conventional 5k + 10 threshold of 40 for six-study analyses, and therefore, this pooled estimate should be interpreted cautiously. In contrast, the PAD-amputation FSN was 93, exceeding the same threshold and supporting greater robustness despite detected asymmetry. For the hypertension-PAD analysis, no significant asymmetry was detected by either test; however, the FSN of 7 falls below the conventional tolerance of 5k + 10 = 40, and this result warrants cautious interpretation.

Narrative Synthesis of Prognostic and Health-System Outcomes of PAD

Formal pooling of prognostic outcome data was not performed owing to substantial heterogeneity in outcome definitions, reporting formats, and follow-up durations across studies. Findings reported by individual included studies are summarized below, with selected study-level data presented in Table 8.

**Table 8:** Narrative Synthesis of Reported Prognostic and Health-System Outcomes of PAD in LMIC DFU Cohorts. ABI: ankle-brachial index; DFU: diabetic foot ulcer; LEA: lower extremity amputation; MAC: medial arterial calcification; OR: odds ratio; PAD: peripheral arterial disease; LMIC: low- and middle-income country Table restricted to studies reporting at least one of amputation or limb-loss outcome, mortality, or hospital stay/resource burden. “Not reported” indicates the outcome was not described by the named study.

Study (year)	Country	PAD prevalence (%)	Reported amputation/limb-loss outcomes	Reported mortality	Reported hospital/resource burden
Hao et al. [5] (2014)	China	70.06	High infection rate (58.9%); major disabling comorbidities in 79% of cohort	Not reported	Prolonged hospitalization; high inpatient resource utilization reported
Ravidas et al. [4] (2020)	India	76.19	All patients with ABI < 0.3 underwent above-knee amputation	Not reported	Wound healing > 120 days in severe lower-limb ischemia
Ede et al. [13] (2018)	Nigeria	61.67	PAD independently predicted major LEA (OR 2.8); 75.6% of amputations were major	10% intra-admission mortality	Not specifically reported
Azhar et al. [6] (2021)	Egypt	43.87	Limb salvage: 82.3% (non-PAD) vs. 48.3% (PAD); PAD was a predictor of non-salvage	Not reported	Not specifically reported
Chen et al. [17] (2024)	China	53.42	44.62% amputation rate in PAD + MAC subgroup; concurrent MAC doubled amputation risk	Increased mortality risk (not quantified)	Not specifically reported
Costa et al. [15] (2017)	Brazil	24.46	2.28-fold higher risk of limb loss in PAD-positive vs. PAD-negative patients	22.2% among amputees vs. 12.0% in overall cohort	Not specifically reported
Nobi et al. [11] (2019)	Bangladesh	34.72	64% of severe ulcers requiring amputation had peripheral vascular disease	Not reported	Mean hospital stay: 48 days (amputation) vs. 16 days (no amputation)

Amputation and limb salvage: PAD was consistently identified as a major determinant of amputation and limb loss across included LMIC studies. Ravidas et al. [4] reported that all patients with ABI < 0.3 underwent above-knee amputation. Ede et al. [13] found that PAD independently predicted major LEA (OR 2.8) and that 75.6% of amputations in their cohort were classified as major. Azhar et al. [6] reported limb salvage rates of 82.3% in patients without PAD compared with 48.3% in those with PAD and identified PAD as the strongest predictor of limb non-salvage (OR 14.9). Chen et al. [17] reported that the combination of PAD and medial arterial calcification (MAC) was associated with a 44.62% amputation rate, with MAC doubling amputation risk beyond PAD alone. Costa et al. [15] reported a 2.28-fold higher risk of limb loss in PAD-positive compared with PAD-negative DFU patients.

Mortality: Mortality data were reported by two included studies. Ede et al. [13] recorded intra-admission mortality of 10% in their DFU cohort. Costa et al. [15] reported mortality of 22.2% among patients who underwent amputation, compared with 12.0% in the overall cohort. The remaining studies did not report mortality outcomes.

Hospital stay and resource burden: Nobi et al. [11] reported a mean hospital stay of 48 days for DFU patients who underwent amputation compared with 16 days for those who did not. Hao et al. [5] described prolonged hospitalization and high inpatient resource utilization in patients with concurrent PAD and DFU. Ravidas et al. [4] reported wound healing times exceeding 120 days in patients with severe lower-limb ischemia. Quantitative resource-utilization data were not consistently reported across the remaining studies.

Discussion

Principal Findings

This systematic review and meta-analysis examined the prevalence, determinants, and clinical consequences of PAD among patients with DFU in LMIC settings, yielding three principal findings. First, PAD is strikingly common in this population: approximately four in 10 patients presenting with a DFU in an LMIC also have PAD. Second, modifiable vascular risk factors, particularly smoking and hypertension, are significantly associated with PAD occurrence, while peripheral neuropathy, longer diabetes duration, and higher ulcer severity show consistent positive associations in a direction that warrants further investigation. Third, and of greatest clinical consequence, PAD substantially amplifies the risk of LEA and is consistently associated with worse limb outcomes, prolonged hospitalization, and greater mortality burden across included settings. Taken together, these findings position PAD not merely as a diagnostic coincidence in patients with DFU, but as a potent comorbidity that shapes limb prognosis and the broader health-system burden of diabetic foot disease in resource-constrained environments.

Interpretation of the Pooled PAD Prevalence

The pooled PAD prevalence of 43.00% conveys a clinically important epidemiological signal: nearly half of all patients attending for DFU care in LMIC settings carry concurrent arterial disease of the lower limb. This burden is substantial irrespective of the diagnostic threshold applied. At the level of a busy diabetic foot or general surgical ward in a district hospital in sub-Saharan Africa, South Asia, or South America, a prevalence of this magnitude implies that vascular compromise is a daily clinical reality rather than an incidental finding. It also underscores that the archetypal DFU in LMIC settings is frequently neuroischemic or primarily ischemic, a disease phenotype that carries materially worse healing prospects and greater amputation risk than purely neuropathic ulceration [21,22].

The high between-study heterogeneity (I² = 84.52%) observed in the primary analysis is both expected and informative. Study-level prevalences ranged from 12.00% to 76.19%, a span that almost certainly reflects genuine between-setting variation rather than statistical artefact alone. Several structural factors plausibly explain this range. Referral pathway effects are paramount: studies conducted at tertiary vascular or diabetic foot centers will recruit patients with more advanced disease and, by extension, higher PAD rates, whereas community-level or primary-care studies tend to capture milder or earlier presentations. Case-mix differences including variation in the proportion of patients with longstanding diabetes, high ulcer severity grades, and concurrent cardiac or renal comorbidity further stratify underlying PAD risk across settings. Diagnostic heterogeneity also matters. Most studies contributing to the primary pool applied ABI < 0.9, one study used TBI < 0.7, and several studies additionally used Doppler ultrasonography, duplex imaging, or CTA where clinically indicated. These differences may have altered PAD detection rates, particularly in patients with MAC, where ABI can be falsely elevated [23]. Geographic heterogeneity in cardiovascular risk profiles, smoking prevalence, hypertension burden, and healthcare-seeking behavior across the nine countries represented also contributes to the observed dispersion. These considerations do not undermine the substantive finding; they contextualize it within the structural realities of LMIC health systems.

The leave-one-out sensitivity analysis demonstrated that the primary estimate of 43.00% was robust to the exclusion of any individual study, with the pooled prevalence ranging from 39.37% to 46.80% across iterations. This stability supports confidence in the central estimate, notwithstanding the heterogeneity.

Interpretation of Risk Factors for PAD in DFU Patients

The pooled analyses identified smoking and hypertension as statistically significant associates of PAD in DFU patients, and the directions of association are biologically coherent. The pooled OR for smoking was 2.3561, indicating that DFU patients with a smoking history had more than twice the odds of concurrent PAD. Smoking is a well-established and mechanistically plausible driver of atherosclerosis and peripheral arterial insufficiency. Nicotine-mediated endothelial dysfunction, oxidative stress, dyslipidemia, and prothrombotic effects converge to accelerate atherogenesis in peripheral vessels [23]. In LMIC populations, where tobacco use frequently begins in early adulthood and pack-year burdens can be substantial, the cumulative vascular impact of smoking on peripheral arterial beds is clinically relevant. The moderate between-study heterogeneity observed in this analysis (I² = 61.91%), together with the statistically significant funnel asymmetry on Egger's test, suggests that some caution is warranted in interpreting the precise magnitude of the pooled estimate. However, Rosenthal’s FSN of 23 was below the conventional 5k + 10 threshold for a six-study analysis, so the smoking-PAD association should be interpreted cautiously despite the statistically significant pooled estimate.

The association between hypertension and PAD yielded a pooled OR of 1.8404, with negligible between-study heterogeneity, the most internally consistent estimate in the entire review. Hypertension contributes to arterial stiffness, endothelial injury, and accelerated atherosclerosis in peripheral vessels, and its co-occurrence with diabetes creates a particularly hostile vascular environment [22]. The consistency of the hypertension-PAD association across six studies from five countries, representing three geographic subregions, strengthens confidence in this finding. At the same time, the small FSN of 7 for this analysis falling below the conventional tolerance level appropriately tempers certainty and calls for replication in larger studies with dedicated hypertension measurement and standardized PAD diagnostic protocols.

Peripheral neuropathy, diabetes duration exceeding 10 years, and higher ulcer severity grades each showed positive associative directions in the non-pooled analyses, though the limited number of contributing studies (k = 3 for each domain) and incomplete harmonization of exposure definitions precluded formal meta-analysis. The neuropathy-PAD association is clinically coherent: peripheral neuropathy impairs protective sensation and autonomic regulation of peripheral vasomotor tone, and its co-occurrence with ischemia defines the neuroischemic foot, a phenotype associated with particularly poor healing and high amputation risk [21]. Longer diabetes duration reflects cumulative glycemic exposure and sustained injury to the vascular endothelium, mechanisms that plausibly accelerate atherogenesis in peripheral beds over time [23]. Higher ulcer severity reflected in higher Wagner grades is likely both a cause and a consequence of ischemia in this population: ischemia impairs wound healing and promotes ulcer progression, while more severe ulcers signal more advanced disease at presentation [24]. The fact that these three factors showed consistent positive signals despite small study numbers reinforces their potential clinical relevance and identifies them as priority exposures for future prospective investigations.

Interpretation of Prognostic Outcomes

The prognostic burden of PAD in DFU patients is among the most consequential findings of this review. The pooled OR for LEA was 4.1371 (95% CI 2.2412-7.6293), with the entire 95% CI lying comfortably above unity, implying that DFU patients with PAD faced more than four times the odds of amputation compared with those without PAD. The 95% CI itself is wide, reflecting moderate between-study heterogeneity (I² = 51.97%), but the direction, magnitude, and precision of the estimate are consistent with a clinically important prognostic relationship. These findings are reinforced by the narrative evidence. Azhar et al. [6] demonstrated a difference in limb salvage rates between PAD-positive and PAD-negative patients. Ravidas et al. [4] noted a near-universal above-knee amputation rate among patients with critical ischemia (ABI < 0.3), while Costa et al. [15] demonstrated a more than twofold higher risk of limb loss in PAD-positive patients. These dramatic differences in limb salvage rates collectively illustrate that PAD fundamentally alters the clinical trajectory of DFU in LMIC settings.

The mortality data, though limited to two studies, also warrant careful consideration. Intra-admission mortality of 10% in a Nigerian DFU cohort [13] and post-amputation mortality of 22.2% in a Brazilian series [15] represent serious clinical outcomes in patients who are frequently of working age. The hospital resource data mean hospital stays approaching 50 days in amputation patients [11], wound healing exceeding four months in severe ischemia [4], and prolonged inpatient resource utilization [5] signal that PAD-complicated DFU imposes a disproportionate burden on health systems that are already under pressure. These observations frame PAD not merely as a vascular diagnosis but as an indicator of advanced disease severity, late healthcare presentation, and the downstream costs of incomplete preventive care.

The 44.62% amputation rate reported by Chen et al. [17] in the subgroup with concurrent PAD and MAC deserves particular attention. This finding suggests that the prognostic impact of PAD in DFU may be further compounded by coexisting calcific arteriopathy, a complication associated with autonomic neuropathy and advanced diabetes that can render ABI-based screening unreliable and revascularization technically challenging. This intersection of ischemia and calcific disease is clinically relevant across LMIC settings, where neither advanced vascular imaging nor interventional revascularization services are uniformly available [25].

Comparison With the Broader Review Literature

The findings of this review are broadly consistent with, and extend, the contemporary review-level literature on PAD and diabetic foot disease. Armstrong et al. [21] in their comprehensive review of DFU described PAD as one of the central pathophysiological contributors to non-healing ulceration and limb loss, emphasizing that ischemic and neuroischemic disease presentations are systematically under-recognized in clinical practice. The pooled prevalence reported in the present review aligns with the expectation of a high PAD burden in hospital-based DFU cohorts and supports the contention that PAD detection must be treated as a routine rather than an exceptional clinical task. Rümenapf et al. [22] similarly highlighted the clinical and diagnostic complexity of PAD in the diabetic foot context, including the limitations of conventional ABI measurement in calcified vessels and the frequent co-occurrence of PAD and neuropathy, a finding echoed by the non-pooled neuropathy data from the present review.

On the question of PAD diagnosis and bedside assessment, the systematic review by Chuter et al. [26] provides an important methodological reference point. Their evaluation of ABI, TBI, and other non-invasive methods highlights that diagnostic accuracy varies by test and patient subgroup, and that standard ABI may underperform in the presence of MAC. This has direct implications for interpreting the prevalence data synthesized in the present review: studies relying on ABI alone, without toe pressure or duplex confirmation, may have underestimated true PAD burden in some settings. The generally consistent pooled estimate observed despite this variation suggests that the finding is substantive, but the need for standardized diagnostic protocols across LMIC research settings remains.

With respect to amputation risk, the prognostic finding of the present review that PAD confers more than a fourfold increase in amputation odds is aligned with the current literature. Xie et al. [27] in their systematic review of prognostic and prediction models for DFU progression to amputation, identified PAD and ischemia-related features as among the most powerful predictors of adverse limb outcomes. Meloni and Vas [28] in their dedicated review of PAD in the diabetic foot, described ischemia as a key driver of delayed wound healing, higher amputation rates, and elevated mortality, conclusions that parallel the narrative and quantitative evidence accumulated here from LMIC settings specifically.

Regarding revascularization and limb salvage, Chuter et al. [25] documented the potential for revascularization to improve limb outcomes, while noting that access to these interventions remains unequal globally. This is particularly pertinent in LMICs. The limb salvage differentials observed in the present review, most clearly illustrated by Azhar et al. [6], may partially reflect the limited availability of endovascular or surgical revascularization options in LMIC settings, a structural determinant of outcome that transcends patient-level risk factors.

Pallin et al. [29], in their scoping review of screening practices for diabetic foot disease, highlighted variability in screening protocols as a system-level barrier to early PAD detection, with implications for care pathways in settings where vascular expertise and diagnostic equipment are constrained. Finally, Aditya et al. [23] contextualized the interplay between ischemia, neuropathy, and infection in driving ulcer severity and outcomes, reinforcing the multifactorial interpretation of PAD risk and progression that runs through the present review.

Implications for Practice and Health Systems in LMICs

The aggregated evidence from this review carries several implications for clinical practice and health-system planning in LMICs, though these should be read as interpretations of observational evidence rather than prescriptive recommendations. The most fundamental observation is that PAD is frequently present at the time of DFU presentation in these settings, and that its presence materially worsens prognosis. This suggests that structured clinical assessment for arterial insufficiency using available bedside tools, most practically ABI measurement, has the potential to stratify patients at an early clinical decision point and inform triage, wound management, and referral planning. Where vascular surgery or endovascular services are geographically or economically inaccessible, the clinical value of PAD identification may be less about triggering formal revascularization and more about supporting realistic wound prognosis, optimizing diabetes and hypertension control, facilitating timely referral when services are available, and enabling appropriate patient and family counseling.

The smoking and hypertension findings, though observational, are consistent with a modifiable vascular risk landscape that is addressable within existing LMIC primary and secondary care structures. Tobacco cessation and blood pressure management are interventions with strong evidence bases, relatively low per-patient costs, and relevance far beyond DFU alone. The consistent positive associations between these factors and PAD in the present review reinforce the importance of integrating cardiovascular risk management into diabetic foot care programs in LMIC settings, not as an add-on, but as a component of holistic management.

The resource burden findings of prolonged hospitalization, high amputation rates, and excess mortality documented across African, Asian, and South American cohorts point to the downstream costs of late PAD detection and inadequate vascular assessment infrastructure. Investing in the capacity to identify PAD early, whether through improved point-of-care diagnostic access, workforce training, or care pathway redesign, is likely to be economically rational in addition to clinically important, though formal health economic analyses in LMIC contexts needed to substantiate this inference.

Strengths and Limitations

This review has several methodological strengths. It brings together evidence from multiple LMICs across Africa, Asia, and South America, regions that are systematically under-represented in high-income country-dominated global reviews of diabetic foot disease and synthesizes findings that collectively span over a decade of observational research. By integrating prevalence data, risk factor analyses, and prognostic outcome evidence within a unified systematic framework, the review provides a more complete picture of the PAD burden in LMIC DFU populations than would be possible from any single study or analytical approach alone. The use of random-effects meta-analysis with REML estimation for all pooled analyses appropriately accounts for genuine between-study heterogeneity and avoids the well-documented underestimation of between-study variance inherent in older DerSimonian-Laird approaches [30]. The structured narrative synthesis for non-poolable outcomes preserves clinically important evidence that would otherwise have been excluded from quantitative pooling. Risk of bias was assessed using design-specific JBI critical appraisal instruments applied across cross-sectional, cohort, and prevalence study designs, providing a nuanced and context-appropriate quality evaluation. Publication bias was formally evaluated for all four meta-analyses, and the sensitivity and robustness of the primary estimate were confirmed through a comprehensive leave-one-out analysis.

These findings should be interpreted in the context of several limitations inherent to the underlying evidence base and the analytical choices made in this review. All included studies were observational in design, and the associations reported, particularly those between risk factors and PAD, and between PAD and amputation, should not be interpreted as causal without the support of prospective, longitudinal, and ideally interventional evidence. Confounding by unmeasured variables, including glycemic control, lipid profiles, medication use, and healthcare access, is plausible across most included studies.

Between-study heterogeneity was high in the primary prevalence meta-analysis and moderate in two of the three secondary analyses, indicating genuine variation across settings, patient populations, and study conditions that a single pooled estimate cannot fully represent. The broad CIs for the primary prevalence estimate and some secondary analyses reflect this variability and appropriately caution against overly precise interpretation. Harmonization of exposure definitions was incomplete across non-pooled analyses: neuropathy was assessed by different clinical and neurophysiological methods, diabetes duration thresholds were not uniform, and ulcer severity was operationalized using different grading scales, precluding formal quantitative synthesis and restricting comparison to directional narrative conclusions.

PAD diagnostic approaches were not uniformly reported across all included studies. The primary prevalence pool was largely based on ABI-defined PAD, although one study used TBI < 0.7; therefore, diagnostic approaches were not completely uniform even within the primary prevalence analysis. Studies contributing to secondary analyses did not always provide explicit PAD diagnostic information in the available source material. ABI-based diagnosis has recognized limitations in the diabetic population, where MAC can produce falsely elevated indices and lead to underdiagnosis [31]; studies not supplementing ABI with TBI or duplex imaging may have systematically underestimated PAD prevalence.

Several outcomes of clinical and health-system importance, including mortality, wound healing time, and hospital resource burden, were reported by only a small number of included studies, precluding formal meta-analysis and limiting the robustness of prognostic conclusions to structured narrative synthesis. Possible small-study effects were detected in the smoking-PAD and PAD-amputation analyses on Egger’s regression. The PAD-amputation result appeared more robust based on its FSN, whereas the smoking-PAD and hypertension-PAD analyses should be interpreted cautiously because their FSN values were below the conventional tolerance threshold. The potential influence of publication bias and selective reporting cannot be entirely excluded in a field where small, single-center LMIC studies predominate. In addition, the included countries were predominantly middle-income rather than low-income economies, and the poorest settings where access to vascular diagnostics and revascularization is most severely constrained were under-represented; the pooled estimates may therefore not fully capture the true burden and consequences of PAD in low-income contexts, and this should be borne in mind when generalizing the findings across the full income spectrum of LMICs. Finally, the number of contributing studies for several secondary and non-pooled analyses was small, constraining statistical precision and limiting the generalizability of subgroup findings.

## Conclusions

PAD is highly prevalent among patients with DFUs in LMICs and is associated with substantially worse limb outcomes, including a markedly elevated risk of LEA, reduced rates of limb salvage, more severe ischemia at presentation, prolonged hospitalization, and excess mortality burden. Smoking and hypertension emerge as the most consistently documented modifiable vascular risk factors in this population, while peripheral neuropathy, longer diabetes duration, and higher ulcer severity show directionally consistent associations that require more rigorous investigation. These findings have implications for how PAD is assessed, documented, and acted upon within diabetic foot care programs in resource-constrained settings. The current body of evidence, though valuable, is characterized by methodological inconsistency in exposure and outcome definitions, reliance on relatively small single-center studies, and limited prospective data. Future research in LMIC settings should prioritize standardized PAD diagnostic protocols, harmonized outcome definitions, prospective cohort designs, and longer follow-up periods to produce evidence that can more reliably inform care pathways and health-system investment in the prevention and management of limb-threatening diabetic foot disease.

## Appendices

Supplemental material 1

Search Queries

PubMed 92

("peripheral arterial disease" OR "peripheral artery disease" OR "peripheral vascular disease" OR "critical limb ischemia" OR "ankle brachial index") AND ("diabetic foot ulcer" OR "diabetic foot" OR "foot ulcer" OR "DFU") AND ("Africa" OR "Asia" OR "Latin America" OR "developing countries" OR "low-income" OR "middle-income" OR "LMIC" OR "India" OR "Nigeria" OR "Ethiopia" OR "Egypt" OR "Brazil" OR "Iran" OR "Pakistan" OR "Bangladesh") AND ("2010"[PDAT] : "2024"[PDAT])

Scopus 214

TITLE-ABS-KEY ( ( "peripheral arterial disease" OR "peripheral artery disease" OR "peripheral vascular disease" OR "critical limb ischemia" OR "ankle brachial index" OR "limb ischemia" ) AND ( "diabetic foot ulcer" OR "diabetic foot" OR "foot ulcer" OR "DFU" OR "diabetic gangrene" OR "lower limb amputation" ) AND ( "Africa" OR "Asia" OR "Latin America" OR "developing country*" OR "low-income" OR "middle-income" OR "LMIC" OR "resource-limited" OR "India" OR "Nigeria" OR "Ethiopia" OR "Egypt" OR "Brazil" OR "Iran" OR "Pakistan" OR "Bangladesh" OR "Indonesia" ) ) AND PUBYEAR > 2009 AND PUBYEAR > 2009 AND PUBYEAR < 2027

Lens.org 242

abstract:("peripheral arterial disease" OR "peripheral artery disease" OR "peripheral vascular disease" OR "critical limb ischemia" OR "ankle brachial index") AND abstract:("diabetic foot" OR "diabetic foot ulcer" OR "foot ulcer" OR "DFU") AND abstract:("Africa" OR "Asia" OR "Latin America" OR "low-income" OR "middle-income" OR "LMIC" OR "developing countr*" OR "India" OR "Nigeria" OR "Ethiopia" OR "Egypt" OR "Brazil" OR "Iran" OR "Pakistan" OR "Bangladesh" OR "Uganda" OR "Kenya" OR "Ghana" OR "Indonesia")

Web of Science 166

TOPIC: ("peripheral arterial disease" OR "peripheral artery disease" OR "peripheral vascular disease" OR "critical limb ischemia" OR "ankle brachial index" OR "limb ischemia") AND TOPIC: ("diabetic foot ulcer" OR "diabetic foot" OR "foot ulcer" OR "DFU" OR "diabetic gangrene" OR "diabetic wound")

Supplemental material 2

**Table 9:** Characteristics of All Unique Studies Included Across Quantitative and Narrative Components. ABI: ankle-brachial index; DFU: diabetic foot ulcer; EMRO: Eastern Mediterranean Regional Office; N: sample size; NR: not reported in available source material; PAD: peripheral arterial disease; SEARO: South-East Asia Regional Office; SSA: sub-Saharan Africa; WPRO: Western Pacific Regional Office WHO regional classifications used for consistency. Where N is marked as derived, the figure was computed from the row totals of 2 × 2 contingency tables provided in the source data. *Studies are listed in chronological order.

Author (year)	Country	Region	Study design	N	PAD diagnostic method	Main exposure/outcome contributed	Results; component(s) contributed to
Ikem et al. [3] (2010)	Nigeria	Africa (SSA)	Prospective comparative	46	ABI < 0.9	PAD prevalence	Primary prevalence meta-analysis
Hao et al. [5] (2014)	China	Asia (WPRO)	Retrospective cohort	1,002	ABI < 0.9 + pulse palpation	PAD prevalence; diabetes duration; ulcer severity	Primary prevalence meta-analysis; additional risk factors (non-pooled)
Costa et al. [15] (2017)	Brazil	South America	Retrospective cohort	654	ABI < 0.9	PAD prevalence; PAD → amputation; prognostic outcomes	Primary prevalence meta-analysis; PAD-amputation meta-analysis; narrative synthesis
Muthiah et al. [8] (2017)	India	Asia (SEARO)	Cross-sectional	150	ABI < 0.9 + Doppler	PAD prevalence; PAD → amputation	Primary prevalence meta-analysis; PAD-amputation meta-analysis
Thulasikumar and Vijayasarathy [19] (2017)	India	Asia (SEARO)	Cross-sectional	100	ABI < 0.9	Smoking; hypertension; peripheral neuropathy	Smoking-PAD meta-analysis; hypertension-PAD meta-analysis; additional risk factors (non-pooled)
Ede et al. [13] (2018)	Nigeria	Africa (SSA)	Cross-sectional	60	ABI < 0.9	PAD prevalence; prognostic outcomes	Primary prevalence meta-analysis; narrative synthesis
Megallaa et al. [16] (2019)	Egypt	Africa (EMRO)	Cross-sectional	180	ABI < 0.9	PAD prevalence	Primary prevalence meta-analysis
Ugwu et al. [12] (2019)	Nigeria	Africa (SSA)	NR	336	NR	PAD → amputation	PAD-amputation meta-analysis
Ravidas et al. [4] (2020)	India	Asia (SEARO)	Prospective observational	105	ABI < 0.9	PAD prevalence; prognostic outcomes	Primary prevalence meta-analysis; narrative synthesis
Nobi et al. [11] (2019)	Bangladesh	Asia (SEARO)	Prospective observational	72	ABI < 0.9 + duplex	PAD prevalence; smoking; PAD → amputation; hypertension; resource burden	Primary prevalence meta-analysis; smoking-PAD meta-analysis; PAD-amputation meta-analysis; hypertension-PAD meta-analysis; narrative synthesis
Janbakhsh et al. [10] (2021)	Iran	Asia (EMRO)	NR	196	NR	Peripheral neuropathy; diabetes duration	Additional risk factors (non-pooled)
Tao et al. [18] (2023)	China	Asia (WPRO)	NR	120	NR	Smoking; hypertension; ulcer severity	Smoking-PAD meta-analysis; hypertension-PAD meta-analysis; additional risk factors (non-pooled)
Azhar et al. [6] (2021)	Pakistan	Asia (SEARO)	NR	392	NR	Smoking; PAD → amputation; hypertension; neuropathy; ulcer severity	Smoking-PAD meta-analysis; PAD-amputation meta-analysis; hypertension-PAD meta-analysis; additional risk factors (non-pooled)
Usman et al. [2] (2023)	Pakistan	Asia (SEARO)	NR	206	NR	Diabetes duration	Additional risk factors (non-pooled)
Abu-Jableh et al. [1] (2024)	Jordan	Asia (EMRO)	Cross-sectional	133	ABI < 0.9	PAD prevalence; smoking; hypertension; limb salvage	Primary prevalence meta-analysis; smoking-PAD meta-analysis; hypertension-PAD meta-analysis; narrative synthesis
Chen et al. [17] (2024)	China	Asia (WPRO)	Prospective cohort	979	ABI < 0.9 + Doppler	PAD prevalence; smoking; PAD → amputation; hypertension; prognostic outcomes	Primary prevalence meta-analysis; smoking-PAD meta-analysis; PAD-amputation meta-analysis; hypertension-PAD meta-analysis; narrative synthesis
Kumar and Sethupathy [7] (2024)	India	Asia (SEARO)	Cross-sectional	100	ABI < 0.9	PAD prevalence	Primary prevalence meta-analysis
Mixed designs	4,831*

Note on Total Unique-Study Count and Participant Total

The 17 unique included studies were identified by compiling all studies that contributed data to any component of the review - primary prevalence meta-analysis, three secondary meta-analyses, additional non-pooled risk factor analyses, and narrative synthesis - and then deduplicating by first author and year so that each study was counted once only, regardless of how many analytical components it contributed to.

The minimum available participant total of 4,831 was derived by summing the sample size of each unique study once only. For studies whose sample size was directly reported in the original source material [1,3-5,7,8,11,13,15-17], the reported figure was used directly. For six studies whose sample size was not separately stated in the source material [2,6,10,12,18,19], the figure was derived from the row and column totals of the 2 × 2 contingency tables available for those studies. Because derived figures are approximations and may not account for any exclusions applied in the original studies, the total is described as a minimum available total rather than a verified enrolled total. No participant was counted more than once: where the same study contributed data to multiple analytical components, its sample size was included in the total only once.

Supplemental material 3

**Table 10:** JBI Risk-of-Bias Assessment of Unique Included Studies. Studies that prospectively or retrospectively followed cohorts to identify predictors of clinical outcomes (e.g., amputation or mortality) were appraised using the JBI Checklist for Cohort Studies. All other studies focusing on the association between candidate risk factors and the prevalence of PAD were appraised using the JBI Checklist for Analytical Cross-Sectional Studies. Overall, nine studies (52.9%) were rated as low risk of bias, reflecting strong participant selection procedures and generally appropriate analytical methods. The remaining eight studies (47.1%) were rated as moderate risk of bias, most often because of incomplete adjustment for confounding or limitations related to convenience hospital-based sampling. No study was rated as high risk of bias, and all identified studies were therefore retained in the final synthesis. ACS: analytical cross-sectional; JBI: Joanna Briggs Institute; PAD: peripheral arterial disease; TBI: toe-brachial index; STROBE: STrengthening the Reporting of OBservational studies in Epidemiology; MEDFUN: Multi-center Evaluation of Diabetic Foot Ulcer in Nigeria. Key: Y: yes; N: no; U: unclear; N/A: not applicable

Author (year)	Country	Study design	JBI tool	Q1	Q2	Q3	Q4	Q5	Q6	Q7	Q8	Q9	Q10	Q11	Overall appraisal	Notes
Ikem et al. [3] (2010)	Nigeria	Prospective cohort	Cohort	Y	Y	Y	U	N	Y	Y	Y	U	U	Y	Moderate	Limited adjustment for confounders in a small sample.
Hao et al. [5] (2014)	China	Retrospective cohort	Cohort	Y	Y	Y	Y	Y	Y	Y	Y	Y	U	Y	Low risk	Robust consecutive enrollment and clear outcome ascertainment.
Costa et al. [15] (2017)	Brazil	Retrospective cohort	Cohort	Y	Y	Y	Y	Y	Y	Y	Y	Y	U	Y	Low risk	Comprehensive multivariable adjustment.
Muthiah et al. [8] (2017)	India	Analytical cross-sectional	ACS	Y	Y	Y	Y	U	N	Y	Y	N/A	N/A	N/A	Moderate	Confounding variables not fully addressed.
Thulasikumar and Vijayasarathy [19] (2017)	India	Analytical cross-sectional	ACS	Y	Y	Y	Y	U	N	Y	Y	N/A	N/A	N/A	Moderate	Hospital-based convenience sample.
Ede et al. [13] (2018)	Nigeria	Analytical cross-sectional	ACS	Y	Y	Y	Y	Y	Y	Y	Y	N/A	N/A	N/A	Low risk	Inclusion of a matched comparison group.
Megallaa et al. [16] (2019)	Egypt	Analytical cross-sectional	ACS	Y	Y	Y	Y	U	N	Y	Y	N/A	N/A	N/A	Moderate	Potential for selection bias in clinic-based recruitment.
Ugwu et al. [12] (2019)	Nigeria	Prospective cohort	Cohort	Y	Y	Y	Y	Y	Y	Y	Y	Y	Y	Y	Low risk	Large multicenter evaluation (MEDFUN).
Ravidas et al. [4] (2020)	India	Prospective cohort	Cohort	Y	Y	Y	Y	U	Y	Y	Y	Y	U	Y	Low risk	Detailed outcome assessment with clear clinical endpoints.
Nobi et al. [11] (2019)	Bangladesh	Prospective cohort	Cohort	Y	Y	Y	Y	U	Y	Y	Y	U	U	Y	Moderate	High exclusion rate from the initial pool.
Janbakhsh et al.[10] (2021)	Iran	Analytical cross-sectional	ACS	Y	Y	Y	Y	U	N	Y	Y	N/A	N/A	N/A	Moderate	Confounding strategy was insufficiently described.
Tao et al. [18] (2023)	China	Analytical cross-sectional	ACS	Y	Y	Y	Y	Y	Y	Y	Y	N/A	N/A	N/A	Low risk	Binary logistic regression was appropriately applied.
Azhar et al. [6] (2021)	Pakistan	Analytical cross-sectional	ACS	Y	Y	Y	Y	Y	Y	Y	Y	N/A	N/A	N/A	Low risk	Objective PAD assessment with clear case definition.
Abu-Jableh et al. [1] (2024)	Jordan	Analytical cross-sectional	ACS	Y	Y	Y	Y	Y	Y	Y	Y	N/A	N/A	N/A	Low risk	Objective TBI and symptom assessment tool used.
Chen et al. [17] (2024)	China	Prospective cohort	Cohort	Y	Y	Y	Y	Y	Y	Y	Y	Y	Y	Y	Low risk	STROBE-compliant study with extended follow-up.
Kumar and Sethupathy [7] (2024)	India	Analytical cross-sectional	ACS	Y	Y	Y	Y	U	N	Y	Y	N/A	N/A	N/A	Moderate	Small sample and limited multivariable risk analysis.
Usman et al. [2] (2023)	Pakistan	Analytical cross-sectional	ACS	Y	Y	Y	Y	U	N	Y	Y	N/A	N/A	N/A	Moderate	Primarily descriptive analysis with limited confounder adjustment.
